# SERS-Based Immunoassay on Ag/ZnO Nanorod Substrates for Detection of CA125 Antigen

**DOI:** 10.1021/acsmeasuresciau.5c00108

**Published:** 2025-12-02

**Authors:** Luis Zamora-Peredo, María Guadalupe Soriano-Rosales, Adriana Baez-Rodríguez, Julián Hernández Torres, Leandro García-González, Marcos Luna Cervantes, Enrique Juárez-Aguilar

**Affiliations:** † Centro de Investigación En Micro Y Nanotecnología, 27870 Universidad Veracruzana, Av. Adolfo Ruiz Cortines 455 Costa Verde, Boca Del Río 94294, México; ‡ Instituto de Ciencias de la Salud, Universidad Veracruzana, Av. Dr. Luis Castelazo Ayala s/n Industrial Animas, Xalapa-Enriquez 91190, México

**Keywords:** Ag/ZnO nanorods, SERS, CA125, immunoassay, cancer, label-free

## Abstract

Several reports have been published on the detection of the carbohydrate antigen 125 (CA125) cancer biomarker, where the immunoassay is completed with a molecule tag that is detected via surface-enhanced Raman scattering (SERS); however, it is still challenging to detect protein biomarkers without a Raman reporter. In this study, a SERS substrate based on zinc oxide nanorods (ZnO NRs) decorated with silver nanoparticles was fabricated, functionalized, and bioconjugated to detect CA125. Functionalization was performed by using an MPA self-assembled monolayer, which was subsequently surface-activated with an EDC/NHS solution. This process was optimized by using Raman measurements to determine the surface protonation of the substrate. The effect of the concentration and incubation time of the CA125 antibodies on the bioconjugation of the substrate were evaluated. SERS detection of CA125 was successfully achieved in a concentration range of 15–1000 U/mL, demonstrating performance comparable to the ELISA approach. A vibration mode at 829 cm^–1^ associated with proline and tyrosine was identified and exhibited excellent linearity with CA125 concentration. A limit of detection (LoD) of 14 U/mL was estimated. This report confirms the potential of Ag/ZnO NR substrates for developing SERS assays for tumor marker detection using a portable Raman spectrometer.

## Graphical abstract

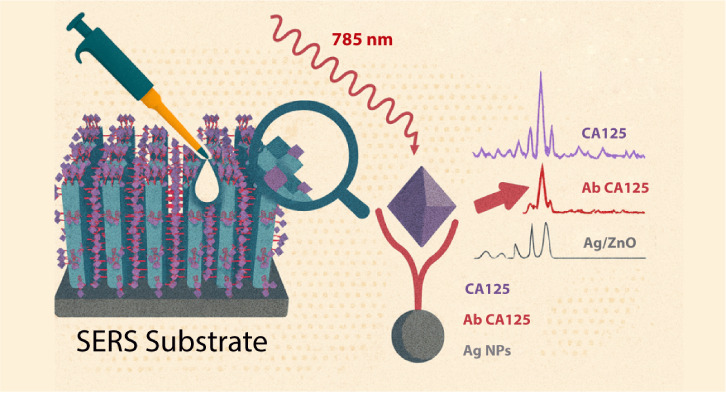

## Introduction

Since its approval by the U.S. Food and Drug Administration (FDA) in 1981, carbohydrate antigen 125 (CA125) has been one of the eight biomarkers that have generated the most significant number of publications^1^ and is mainly associated with the diagnosis of epithelial ovarian cancer (OC).^2–5^ Although CA125 is not recommended for early-stage detection due to its limitations in terms of sensitivity and specificity,^5^ it remains a valuable biomarker for diagnosing advanced-stage ovarian tumors (types I and II),^4^ for tracking the efficacy of chemotherapy, and for assessing patient prognosis.^6^ In order to enhance sensitivity, CA125 has been proposed for use in combination with other biomarkers, such as HE4, CEA, VCAM-1, Ag-AAb, and osteopontin, for the early detection of ovarian cancer,^5–8^ as well as with biomarkers such as CEA, LRG1, REG3A, THBS2, TIMP1, TNRFSF1A, and CA19-9 for the detection of pancreatic cancer.^9^ Several clinical assay strategies have recently focused on multiplexed biomarker testing to improve cancer detection accuracy.^10^ Notably, serum complexes of the HE4 antigen–autoantibody (Ag–AAb) have been shown to complement CA125, significantly increasing the sensitivity of early-stage ovarian cancer detection when used in combination.^5–11^ Furthermore, combining CA125 with other biomarkers is widely recognized as an effective strategy to improve specificity and overall diagnostic performance in ovarian cancer detection.^12^ Moreover, elevated CA125 levels have also been linked to other gynecological cancers, which include cervical,^13^ endometrial,^14^ and fallopian tube cancers;^15^ even to nongynecological cancers like stomach,^16^ breast,^17^ pancreatic,^18^ thyroid,^19^ colorectal,^20^ and lung.^21^ In addition, CA125 has also been associated with noncancerous conditions, such as heart failure.^22,23^ Subsequently, it is easy to understand why there is considerable research into developing new protocols, clinical trials, and new technology for the more efficient detection of CA125.

Surface-enhanced Raman spectroscopy (SERS) biosensors are analytical platforms designed to detect and characterize biomolecules by exploiting the significant amplification of Raman signals produced near plasmonic nanostructures. In general, these systems integrate metallic nanostructures, which are most commonly based on Au or Ag, with specific biorecognition elements such as antibodies, aptamers, or peptides, enabling highly sensitive and selective detection of target analytes through their vibrational fingerprints.^24,25^ Depending on the application, SERS biosensors can be fabricated in various architectures, including colloidal nanoparticles, nanostructured thin films, lithographically defined arrays, or hybrid semiconductor–metal composites,^26–28^ and have been successfully applied to the detection of proteins, nucleic acids, metabolites, and other biomolecules relevant to clinical diagnostics.^24,28–30^ Additionally, considering that the SERS intensity is determined by the distance between the analyte and the metallic surface,^31^ the biomarker under study must be immobilized between metal nanoparticles to obtain better SERS signals.

Several strategies have been reported for cancer biomarker detection using SERS. For example, Zhang et al.^32^ designed a SERS-active chip with polystyrene colloid sphere arrays@Ag/SiO_2_/Ag shell (PS@Ag/SiO2/Ag) for detecting alpha-fetoprotein (AFP), where they used 5,5-dithiobis (succinimidyl-2-nitrobenzoate) (DSNB) as a coupling agent between the silver surface and the AFP antibody before capturing the AFP antigen. They observed vibrational modes associated with DSNB and only one Raman mode at 1390 cm^–1^, the intensity of which increases as the concentration of the AFP antigen increases. The range of AFP concentrations evaluated was 0–8 ng/mL, and the LoD was determined to be 0.078 ng/mL. Recently, Xu et al.^33^ utilized DSNB-functionalized magnetic nanoparticles (Fe_3_O_4_@Au MNPs) for the sensitive detection of the colorectal cancer (CRC) protein biomarker carbohydrate antigen 19-9 (CA19-9). Similarly, they observed vibrational modes associated with DSNB and Raman modes at 1392 cm^–1^, whose intensity increases as the concentration of the CA19-9 antigen increases. They evaluated different CA19-9 concentrations (0.01, 0.1, 1, 10, 100, and 1000 U/mL) and calculated an LoD of 0.27 U/mL. In both reports, vibrational modes observed in the SERS spectra were associated with the linker molecule (DSNB) because it is located within the hot spots, and any Raman mode was directly associated with the complex antibody–antigen. TunÇ et al.^34^ fabricated a SERS substrate with gold nanoparticles (AuNPs) that were functionalized with MPA and EDC/NHS solutions to bioconjugate them with CA125 antibodies (Ab CA125) and subsequently capture CA125 antigens. Raman bands around 510, 650, 740, 930, 1130, 1240, 1265, 1350, 1450, 1650, and 1670 cm^–1^, respectively, assigned to S–S bridge, C–S stretching, C_α_–C stretching, N–C_α_–C stretching, C–N stretching, amide III of β strands, α helices, C–C_α_–H bending, C–H bending, α helices, and amide I of β strands were observed. The SERS spectra observed after incubating Ab CA125 and CA125 showed similar Raman peaks with slight intensity changes caused by their varying separation distances from the substrate hot spots. However, studies focusing on the molecular-level characterization of the CA125–antibody complex remain very limited.

Achieving high selectivity is essential for the clinical translation of SERS biosensors, as it ensures accurate recognition of the target biomarker within complex biological environments. In many SERS immunoassays, selectivity is achieved through a sandwich configuration in which the target antigen is captured between two antibodies—a capture antibody immobilized on the substrate and a detection antibody conjugated to plasmonic nanoparticles—enabling both molecular recognition and signal amplification.^35^ Recent studies have shown that using monoclonal antibodies as capture elements provides particular recognition of target biomarkers, significantly reducing cross-reactivity with structurally similar proteins in complex biological samples such as serum.^36,37^ This selective recognition facilitates the localization of antigen molecules at plasmonic hot spots, where the electromagnetic field is maximized, thereby enhancing the Raman signal intensity. Additionally, monoclonal antibodies offer consistent epitope specificity and binding affinity, which improve the reproducibility and reliability of the detection process.^38,39^ These molecular-level characteristics are crucial for obtaining meaningful vibrational information from the antibody–antigen complex and for improving the diagnostic relevance of SERS-based immunoassays.

In recent years, plasmonic semiconductor–metal nanohybrids have emerged as a powerful class of SERS substrates, combining the intense electromagnetic enhancement provided by noble-metal nanoparticles with the additional charge-transfer capabilities and tunable properties of semiconductor scaffolds. Metal–semiconductor heterostructures leverage the interplay between plasmonic excitation and interfacial electron transfer to significantly boost Raman enhancement beyond what pure metallic systems can achieve.^28,40,41^ Such architectures—typically Au or Ag nanoparticles anchored on semiconductor supports like ZnO, TiO_2_, or Si—offer distinct advantages, including enhanced charge transfer efficiency, customizable optical responses, improved chemical stability, and the formation of additional plasmonic hot spots through optimized metal–semiconductor coupling.^42–45^ As a result, they have found wide applications in fields such as ultrasensitive molecular sensing, biomolecular detection, photocatalysis monitoring, environmental analysis, and point-of-care diagnostics. Among these semiconductor materials, ZnO is particularly compelling due to its high electron mobility, strong excitonic effects, facile growth into one-dimensional morphologies, and excellent chemical compatibility with plasmonic metals.^46–49^ These combined characteristics make ZnO-based plasmonic nanohybrids a promising strategy for enhancing Raman scattering efficiency and expanding the capabilities of SERS-based biosensing platforms.

On the other hand, ZnO nanorod-based SERS substrates (ZnO NRs), where metallic nanoparticles are uniformly distributed throughout a three-dimensional volume, have been proposed to control the 3D distribution of hot spots, which has enabled the achievement of high SERS sensitivity and repeatability.^50–52^ However, most of these strategies still rely on reporter molecules or linkers, resulting in Raman signals that provide only indirect evidence of antigen recognition. Additionally, conventional two-dimensional plasmonic platforms often offer limited hot-spot accessibility for large protein biomarkers, leading to poor sensitivity and reduced spectral information content.

In this study, we address these limitations by engineering a three-dimensional Ag/ZnO nanorod SERS platform that combines the advantages of plasmonic nanohybrids with the molecular specificity of monoclonal anti-CA125 antibodies. The 3D architecture ensures a uniform distribution of plasmonic hot spots throughout the substrate volume, enhancing the interaction between biomolecules and the metallic surface and increasing the probability of molecular recognition events occurring within high-field regions. Under these optimized conditions, the detected Raman bands originate directly from the antibody–antigen complex, providing molecular-level vibrational information associated with specific amino acid residues involved in the recognition process. This strategy not only enables sensitive and selective detection of CA125 within clinically relevant concentration ranges but also enhances the biochemical information content of the Raman spectra, paving the way for more reliable and application-oriented SERS-based immunoassays for cancer biomarker detection.

Beyond addressing these fundamental limitations, the approach presented here offers several practical advantages for future clinical applications. By eliminating the need for external Raman labels or coupling linkers, the assay simplifies the detection process, reduces preparation time and cost, and minimizes sources of variability. Moreover, the ability to obtain molecular-level vibrational information directly from the antibody–antigen complex could support not only detection but also structural or compositional biomarker analysis. These features position the proposed platform as a promising foundation for next-generation diagnostic tools capable of operating in real biological environments.

## Materials and Methods

### Materials

Zinc nitrate hexahydrate (Zn­(NO_3_)_2_·_6_H_2_O), hexamine (C_6_H_12_N_4_), hexamethylenetetramine (HTMA), silver nitrate (AgNO_3_), 3-mercaptopropionic acid (MPA), *N*-hydroxysuccinimide (NHS), 1-ethyl-3-(3-(dimethylamino)­propyl)­carbodiimide (EDC), phosphate-buffered saline (PBS), and the Human Carbohydrate Antigen 125 Mucin-16 ELISA kit were the precursors used, all purchased from Sigma-Aldrich. Human CA125/MUC16 Monoclonal Antibody (Ab CA125) was purchased from R&D Systems, a Bio-Techne brand company.

### Ag/ZnO NR Substrates

As shown in Figure 1, Corning glass slides were coated with ZnO nanoparticles using spray pyrolysis at 400 °C. Subsequently, ZnO NRs were grown in a chemical bath for 6 h at 96 °C using zinc nitrate hexahydrate and hexamine as precursors. The samples were then washed with deionized water and dried at room temperature. For AgNPs’ decoration, ZnO NRs were placed in a solution containing 0.01 M silver nitrate, ethanol, and distilled water. Then, AgNPs were photoreduced over ZnO nanorods with a 375 nm laser for 15 min. After that, the samples were rinsed with deionized water. The detailed methodology is described in the previous report.^54^

**Figure 1:**
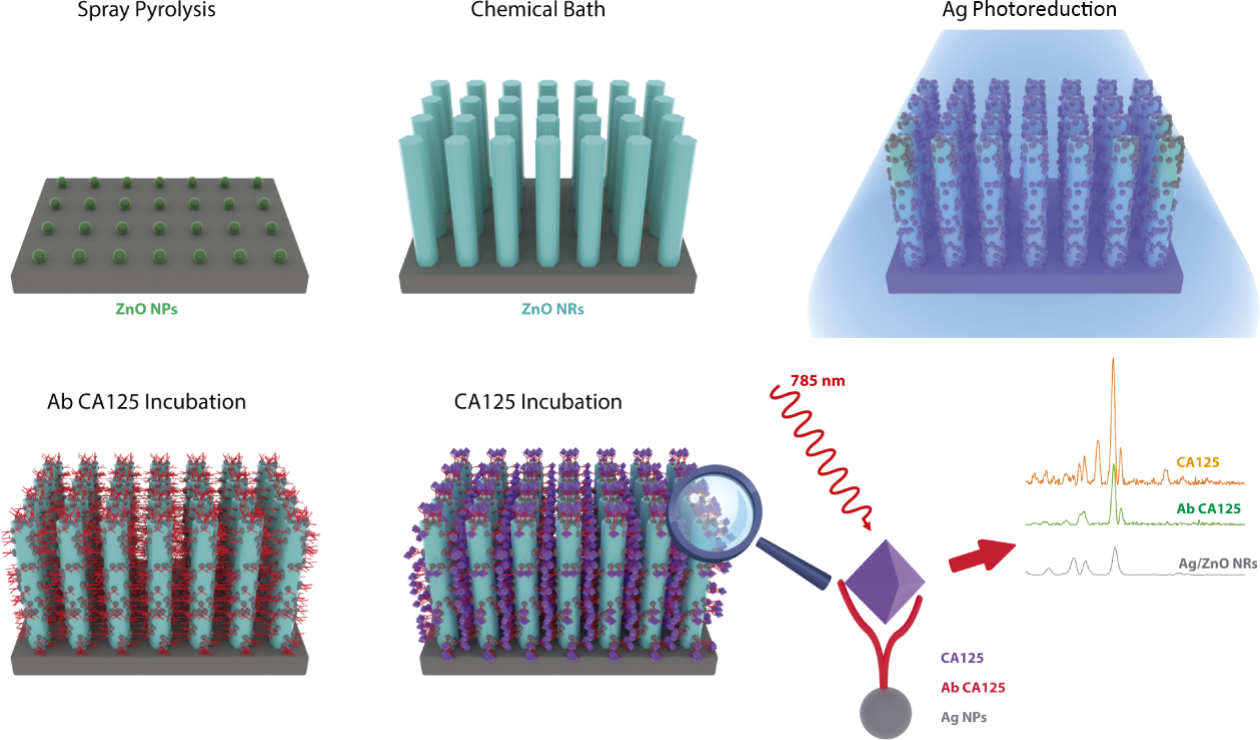
Scheme of the fabrication process of the 3D SERS substrate evaluated in this work.

### Functionalization with MPA and EDC/NHS

The Ag/ZnO NR substrate was prepared by forming different self-assembled monolayers (SAMs) of functional thiols on the AgNPs’ surfaces with a 10 mM MPA aqueous solution. 5 mL of MPA stock solution was added to the substrate to evaluate different incubation times. The substrates were then rinsed twice with deionized water and dried at room temperature.

The terminal carboxyl group of the thiol activation process was carried out in a PBS solution of NHS (100 mM) and EDC (50, 100, and 150 mM). The substrates were immersed in the solution for 1 h to promote the SAM layer growth. The substrates were thoroughly washed with PBS to remove unreacted reagents from the substrate surface.

### CA125 Immunoassays

CA125 antibodies were immobilized on the surface-activated substrate by dripping 100 μL of a solution containing 0.005 mg/mL CA125 antibodies and incubating for 60 min. They were then rinsed four times with a wash solution. A 1% blocking BSA solution (prepared by dissolving BSA in PBS buffer) was used to block the unreacted MPA molecules on the substrates. Next, a 100 μL solution with different concentrations of CA125 antigen (15, 44, 133, 400, and 1000 U/mL) was added dropwise to Ag/ZnO NR substrates and incubated at room temperature for 60 min. Three independent replicates were prepared to ensure the reproducibility of the results.

### Substrate Characterization and SERS Detection

The Ag/ZnO NR substrates were characterized by field-emission scanning electron microscopy (SEM, JEOL, JSM 7600F) to examine the surface morphologies before and after AgNPs photoreduction. The chemical composition and structural properties were analyzed by using a confocal Raman microscope (benchtop, Thermo Scientific, DRX) equipped with a 532 nm laser at a power of 10 mW.

Considering that near-infrared (NIR) excitation reduces fluorescence and photodamage while allowing deeper sample penetration, SERS measurements were performed with a portable Raman system (Ocean Insight),^53^ which includes a QE Pro spectrometer, a 785 nm laser, and a fiber-optic probe. The Raman system has a nominal laser power of 140 mW; however, all measurements were performed at 0.6 of this power (approximately 84 mW) with an integration time of 3 s. The homogeneity of the SERS substrates was evaluated by SERS mapping (benchtop Raman) of a 25 μm × 30 μm area (5 × 6 points) using 10 μL of rhodamine 6G (R6G) at a concentration of 1 × 10^–3^ M prepared with deionized water.

## Results and Discussion

### SERS Substrate Optimization

Figure 2a shows the SEM images of the ZnO NRs with an average diameter of 130 ± 12 nm, a rod density of 25.6 ± 3 rods/μm,^2^ and a hexagonal morphology. Figure 2b shows the ZnO NRs covered by AgNPs obtained after 15 min of photoreduction. More details about the synthesis of the AgNPs/ZnO NR substrate can be found in our previous reports.^54^ It is well accepted that SERS substrates must be optimized by varying the density of AgNPs on the NR surface because, when there are few particles, the hot spots cannot be obtained. On the other hand, when the surface is excessively coated with AgNPs, the SERS intensity decreases.^50^ Figure 2c shows the Raman spectra acquired with the benchtop Raman system of ZnO NRs, where peaks at 99, 333, 378, 437, and 570 cm^–1^, respectively, are associated with the *E*_*2L*_, *E*_*2H*_–*E*_*2L,*_ *E*_*2H*_, A_1TO_, and A_1LO_ vibration modes of hexagonal ZnO.^56^ When the ZnO NRs are covered with AgNPs, the Raman spectra exhibit a broadened peak around 225 cm^–1^, associated with the Ag–O bond,^57^ and the *E*_*2L*_ and *E*_*2H*_ modes are slightly observable, as indicated by the red line in Figure 2d.

**Figure 2:**
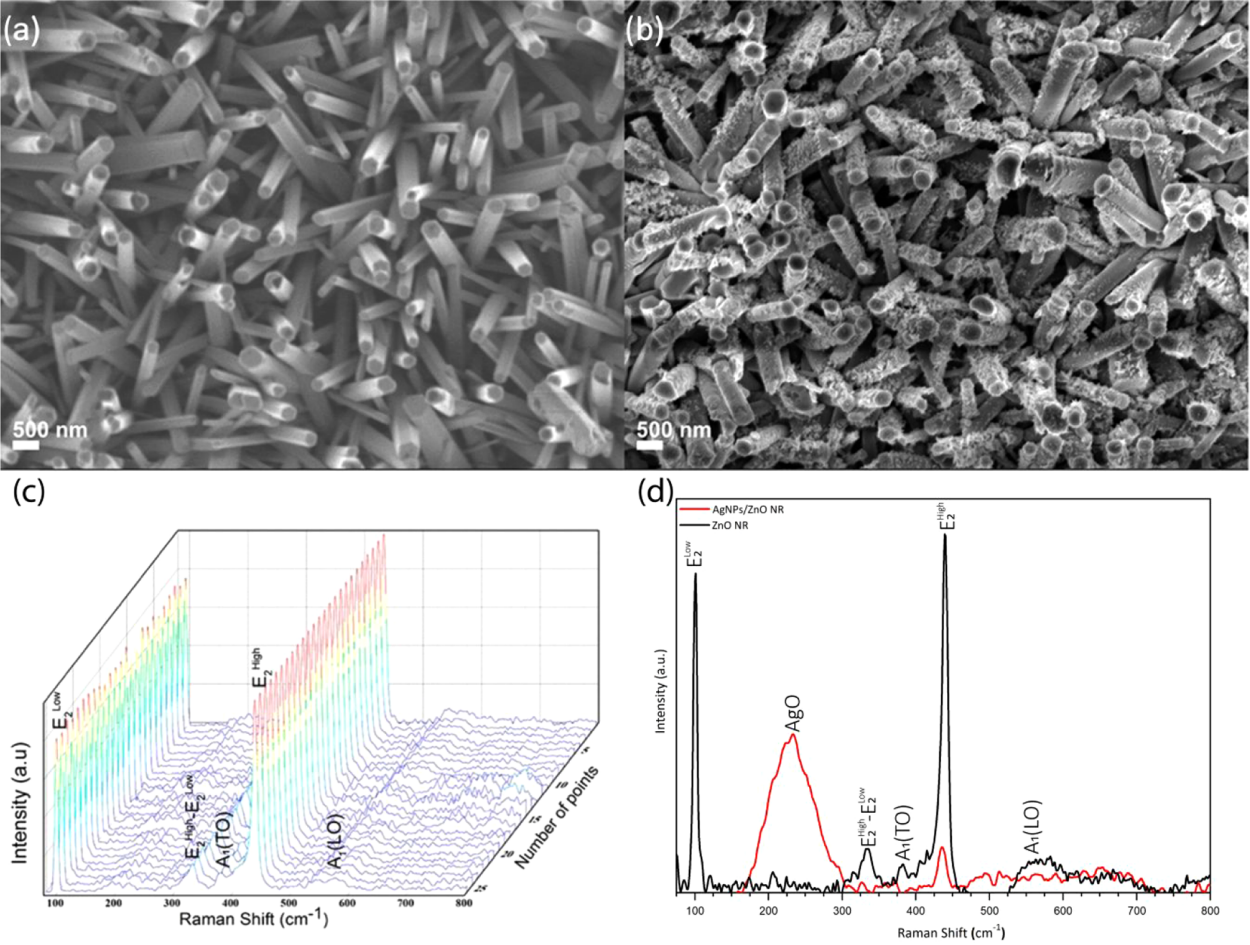
(a,b) SEM images and (c,d) Raman spectra acquired with the benchtop Raman system of ZnO NRs before and after AgNPs photoreduction.

SERS measurements of an aqueous solution with 1 × 10^–3^ M rhodamine 6G (R6G) dropped on substrates with different Ag photoreduction times were performed to explore the enhanced capacity of the substrates. Figure 3a shows the SERS spectra obtained for substrates with Ag photoreduction times between 5 and 30 min, where vibrational modes at 611, 771, 923, 1185, 1308, 1365, 1514, and 1648 cm^–1^ associated with R6G were observed.^50^ Figure 3b shows the intensity behavior of four characteristic modes of the R6G molecule located at 611, 1365, 1514, and 1651 cm^–1^ associated with the vibration of C–C bonds in the xanthene ring,^58^ where it is evident that the SERS intensity had a maximum value at a photoreduction time of 15 min. The intrinsic SERS performance of this substrate was comprehensively evaluated in our previous study,^55^ where a limit of detection of 1 × 10^–8^ M for R6G was reported under comparable experimental conditions.^51^

**Figure 3:**
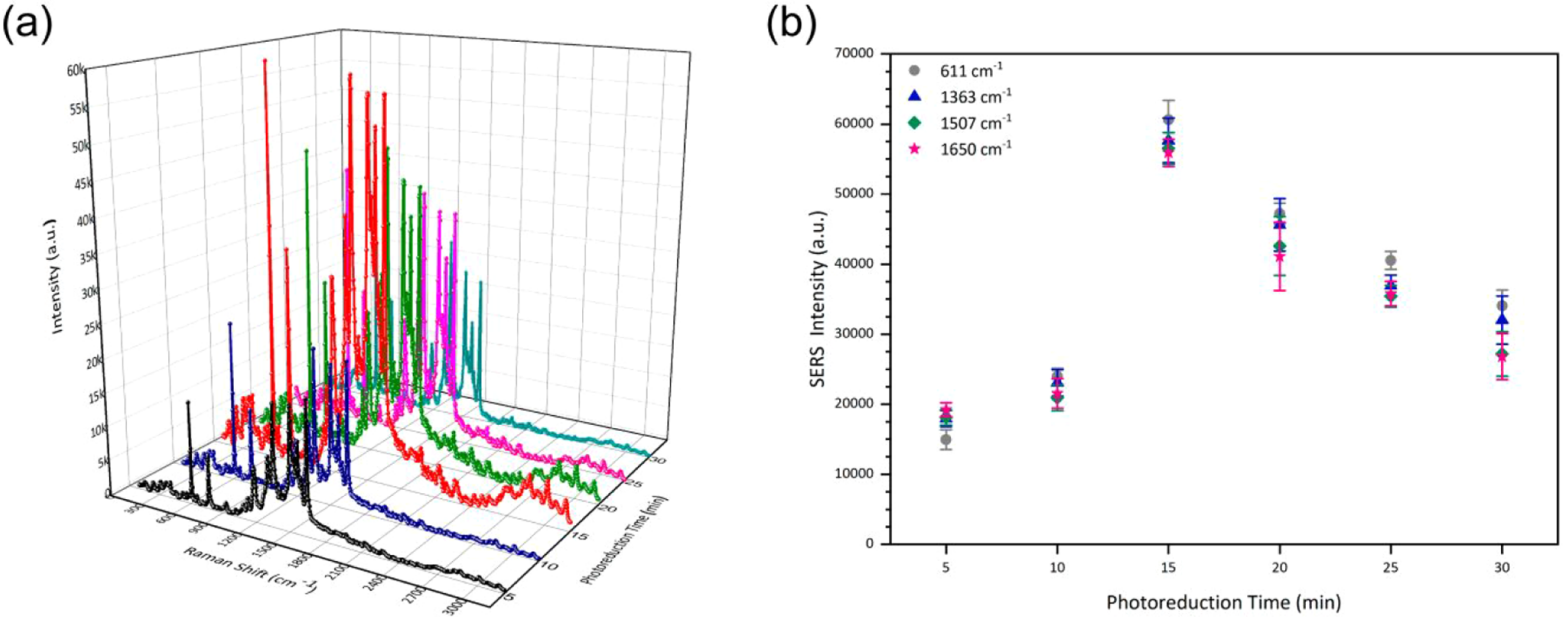
(a) SERS spectra of 1 × 10^–3^ M R6G acquired with the portable Raman system on Ag/ZnO nanorods prepared with different Ag photoreduction times (5–30 min). (b) Intensity evolution of selected Raman bands as a function of the photoreduction time, showing maximum enhancement at 15 min.

### SERS Substrate Bioconjugation

Figure 4a shows the Raman spectra obtained from an aqueous solution of 1 × 10^–2^ M MPA with an incubation time between 0.5 and 8 h, in the range from 400 to 1100 cm^–1^. Four peaks were observed at 504, 663, 745, and 943 cm^–1^, which are characteristic vibration modes of MPA^59,60^ The bands located at 663 and 745 cm^–1^ are assigned to the vibration of the C_1_–C_2_ bond of the Gauche and Anti conformations of the MPA. The Gauche conformation was observed to have a more significant presence in the self-assembled layer of MPA. The most intense mode at 943 cm^–1^, which originates from the deprotonated carboxyl groups, was assigned to the stretching vibration of C–COO.^61^ Figure 4b shows the Raman intensity behavior of the Gauche (G), Anti (A), and C–COO (COO) modes versus incubation time, where it is observed that the three modes exhibit similar behavior and reach their maximum intensity within 1.5 h.

**Figure 4:**
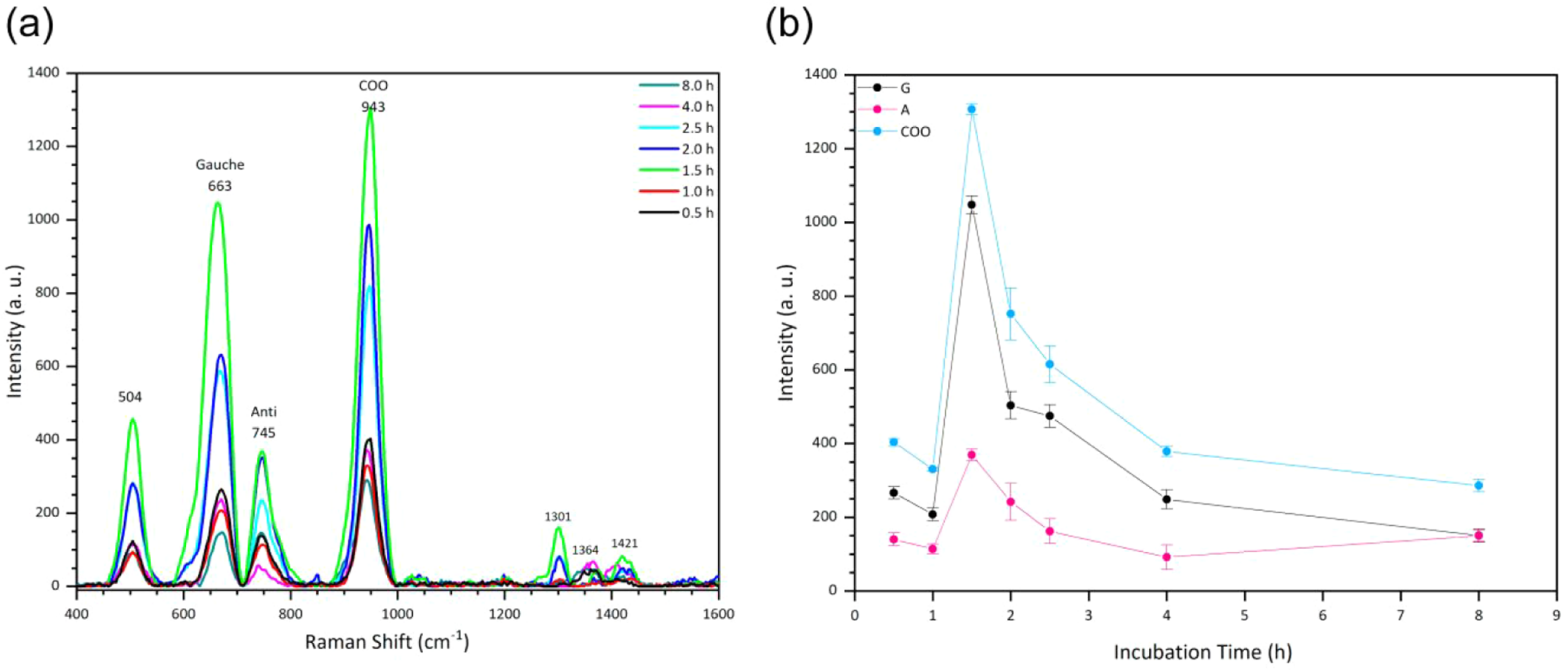
(a) Raman spectra of MPA aqueous solutions incubated for 0.5–8 h on Ag/ZnO NRs acquired with the portable Raman system; (b) Raman intensity behavior of G, A, and COO vibration modes.

To optimize CA125 antibody immobilization on the MPA monolayer surface, an activation treatment using an EDC/NHS solution was performed to form amide covalent bonds between Ab CA125 and the MPA monolayer. Figure 5a shows the Raman spectra after 1 h of incubation of the three EDC/NHS solutions on MPA/Ag/ZnO NRs. The EDC concentration was kept constant at 100 mM, and the NHS concentration was varied between 50, 100, and 150 mM. Figure 5b shows the Raman intensity behavior of the G, A, and COO peaks, where the intensities of the G and A modes decrease as the NHS concentration increases and the A peak intensity does not show similar behavior. This suggests that MPA molecules were ordered perpendicular to the silver surface due to H-bond formation between the protons of secondary amides and the carbonyl groups of neighboring molecules.^62^ In agreement with this finding, the position of the COO peak exhibited a blue shift when the NHS concentration increased, as shown by the green line in Figure 5b.

**Figure 5:**
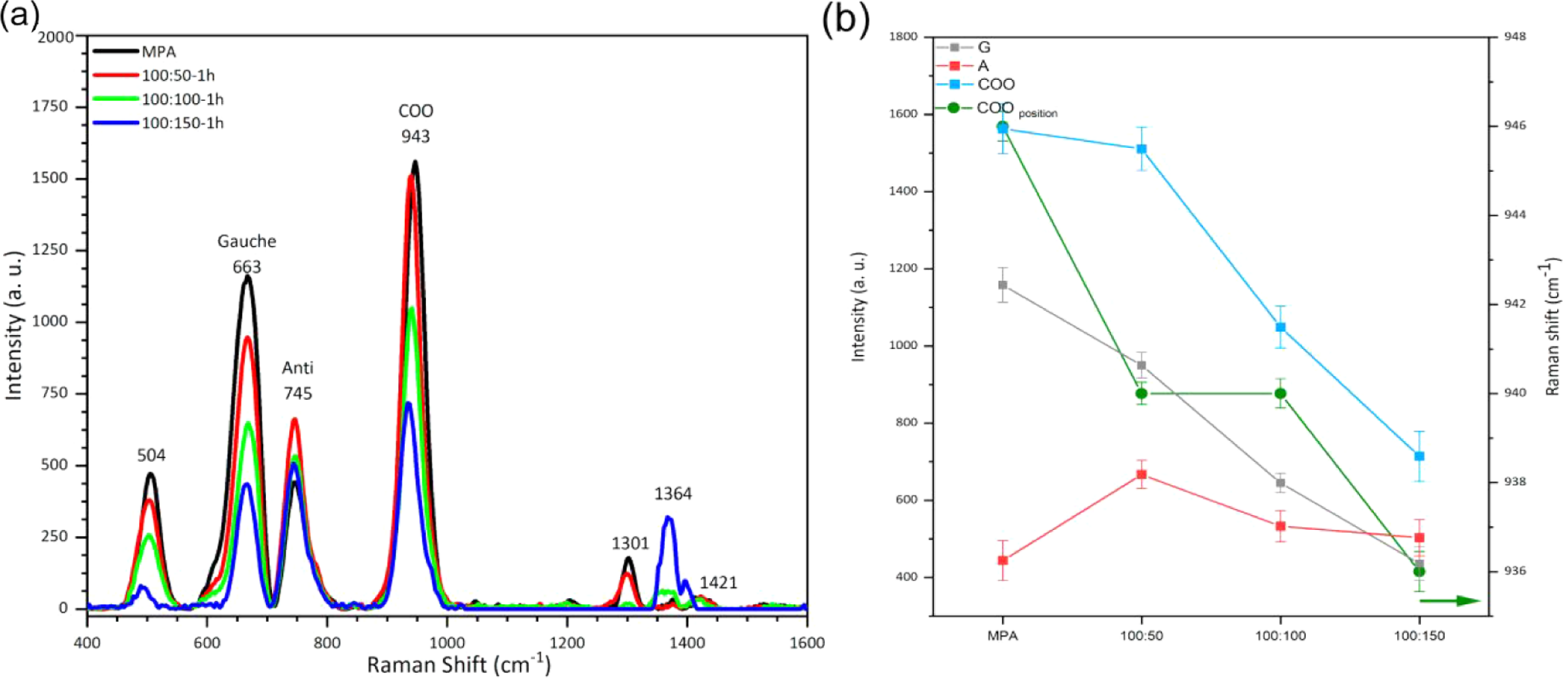
(a) SERS spectra of MPA after 1 h of incubation in EDC/NHS solution with three concentration rates acquired with the portable Raman system and (b) the intensity behavior of G, A, and COO peaks. The green line is the Raman shift of the COO peak.

### Antibody–Antigen SERS Study

Figure 6 shows the SERS spectra of the Ab CA125 (light green line) and CA125 antigen (dark green line) after they were consecutively incubated on an MPA-EDC/NHS-functionalized Ag/ZnO NRs substrate. The SERS spectrum of 0.005 mg/mL Ab CA125 has five vibration modes at 487, 620, 705, 740, 930, and 980 cm^–1^. The intensity of modes at 740 and 930 cm^–1^ is influenced by the intensity of 749 and 940 cm^–1^ modes from the functionalized Ag/ZnO NR substrate (gray line). However, the modes at 487, 620, 705, and 980 cm^–1^ are associated with anchored CA125 antibodies. The new peaks originate due to the amino acids present in antibodies. Considering its similar structure and the 3D spatial distribution, it is challenging to assign each peak to a specific amino acid. The homogeneity of the functionalized substrate was evaluated by depositing the antibody at a concentration of 0.01 mg/mL, as shown in Figure S4.

**Figure 6:**
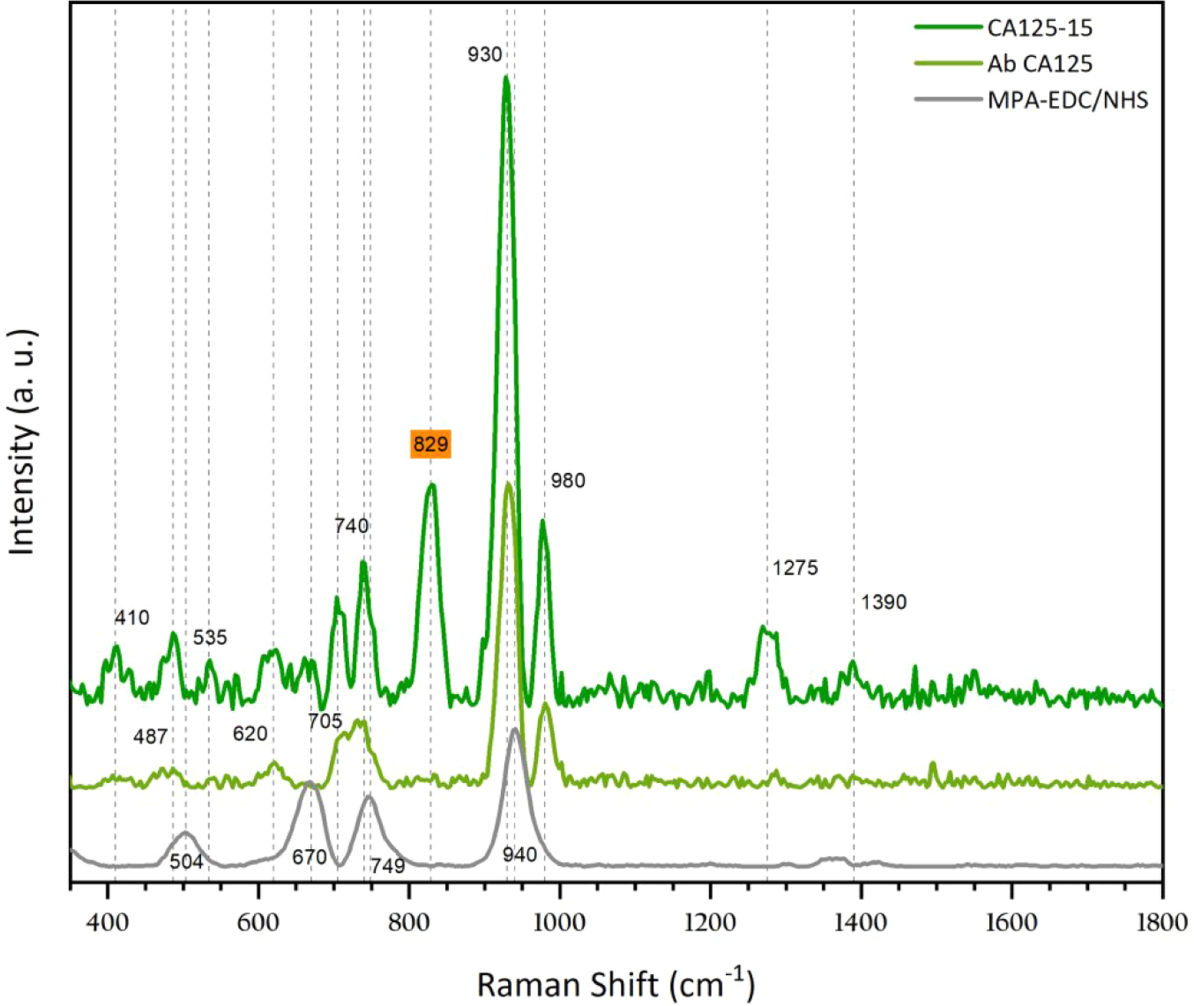
SERS spectra after sequentially incubating 0.005 mg/mL CA125 antibodies (Ab CA125) and 15 U/mL CA125 antigens (CA125-15) on a functionalized Ag/ZnO NR substrate.

Table 1 identifies the 20 amino acids frequently found in antibodies that could originate the Raman peaks observed after Ab CA125 incubation, considering the wavenumbers reported in other studies.^63–65^ The blue color intensity is associated with the Raman intensity observed. Three criteria could be helpful to identify the amino acids: 1) consider the percentage of each amino acid in the crystal structure of rabbit antibodies (used for this study), 2) the best match with the wavenumber observed (±10 cm^–1^), and 3) identify the amino acid whose reported wavenumber matches with three or more observed Raman peaks. Another determining parameter for the SERS intensity is the distance between amino acids and silver particles, as discussed above; however, this is a challenge to evaluate due to the complex 3D SERS substrate configuration, which is beyond the scope of this work.

**Table 1:**
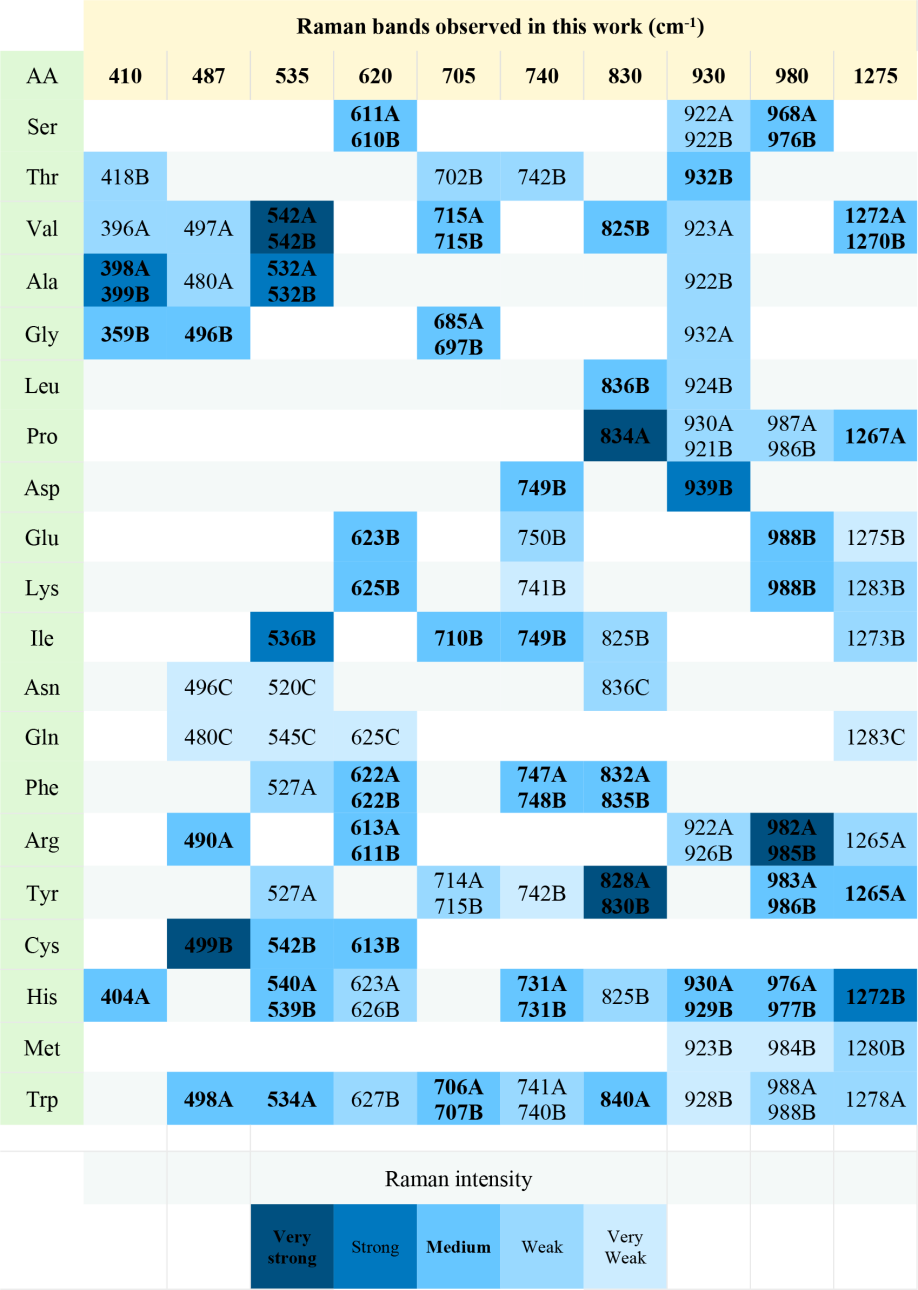
Amino Acids That Could Cause the Raman Bands Observed in This Work, According to the Intensity (Blue Color Level) and Wavenumber Reported by References A,^63^ B,^64^ and C^65^

Completing the SERS immunoassay, when a 15 U/mL CA125 antigen is incubated on the SERS substrate with the Ab CA125, the vibrational modes located at 410, 535, 705, 740, 829, 930, 980, 1275, and 1390 cm^–1^ are observable, as shown by the dark green line in Figure 6. The peaks located at 829, 1275, and 1390 cm^–1^ are the most clearly identifiable. Focusing on the 829 cm^–1^ peak, it is evident from Table 1 that several amino acids are potential sources of this peak; however, proline and tyrosine exhibit the strongest Raman intensity at this wavenumber. Additionally, it was possible to identify six coincident vibrational modes for tyrosine and four for proline, which suggests that both could be the main cause of the 829 cm^–1^ vibration mode.

Figure 7 shows the SERS spectra obtained from liquid solutions with 15, 44, 133, 400, and 1000 U/mL of the CA125 antigen (similar values to those used for ELISA) where we can see that the intensity of the vibrational modes at 410, 473, 535, 620, 705, and 980 cm^–1^ increases as the concentration increases up to 133 U/mL and then decreases for 400 and 1000 U/mL samples. For the two highest concentrations, other notable Raman peaks were observed at 487, 665, 740, 1275, 1370, 1565, and 1570 cm^–1^. However, for all concentrations, only the modes at 829 and 930 cm^–1^ exhibited a progressively increasing SERS intensity as the concentration of CA125 increased, suggesting that proline and tyrosine were systematically located between Ag NPs, where a hotspot exists. The other amino acids did not find good plasmonic resonance in the 3D SERS substrate.

**Figure 7:**
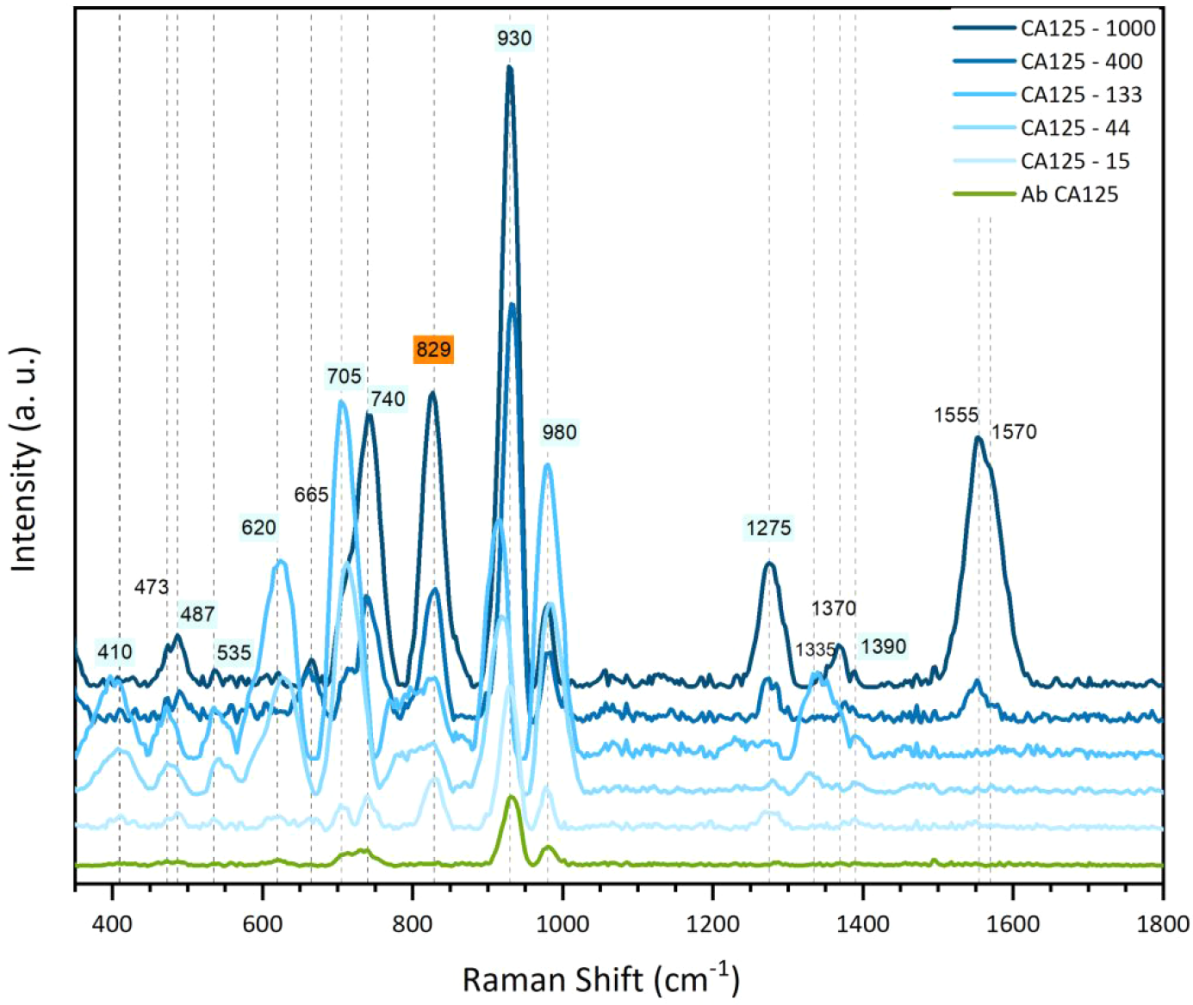
SERS spectra of solutions with different CA125 concentrations.

The mechanism underlying the SERS detection of CA125 in our Ag/ZnO nanorod platform involves the combined action of electromagnetic and chemical enhancement processes, both of which are essential to the observed spectral response of the antibody–antigen complex.^66–68^ The three-dimensional architecture of the Ag/ZnO substrate enables a high density of localized surface plasmon resonances at interparticle junctions and at the metal–semiconductor interface, concentrating the electromagnetic field in highly confined regions^69,70^ In these regions, the local field can be intensified by several orders of magnitude, leading to substantial amplification of the vibrational signals of nearby molecules. Moreover, 3D platforms extend the generation of hot spots beyond the surface plane and into the volume, thereby improving optical field utilization and enhancing signal collection efficiency.

When monoclonal antibodies immobilized on the substrate selectively capture CA125 antigens, the resulting antibody–antigen complexes are positioned within or near these intense near fields, resulting in strong amplification of their vibrational signatures.^71^ This spatial localization is particularly critical for large biomolecules such as CA125, whose size, conformational flexibility, and steric constraints may restrict their approach and effective coupling with plasmonic surfaces in conventional 2D platforms. The 3D nanorod architecture helps overcome these limitations by allowing different regions of the antibody–antigen complex to interact with multiple high-field regions, thereby increasing the probability of effective field–molecule coupling.^72^

In addition to the electromagnetic contribution, charge transfer (CT) between the ZnO semiconductor and the adsorbed biomolecules further enhances the Raman signal.^3,73,74^ The Ag–ZnO interface can facilitate electron exchange with molecular orbitals of the bound antibody–antigen complex, transiently altering their polarizability and Raman scattering cross sections.^75^ These chemical effects, combined with structural rearrangements induced by epitope–paratope recognition, can lead to variations in intensity or even slight shifts in the frequency of specific vibrational bands. In protein SERS studies, selective enhancement of aromatic amino acid residues is often observed due to their stronger coupling with local electromagnetic fields.^76,77^ Indeed, bulky side chains can suppress or modulate specific backbone modes (e.g., amide I) when steric interactions affect adsorption geometry.^78^ Residues such as tyrosine and proline, when brought within nanometer proximity to plasmonic surfaces, have been shown to yield pronounced Raman signals—proline can be distinguished even in single-molecule SERS experiments,^79^ while tyrosine has been detected with ultrasensitive enhancement in plasmonic environments.^80^

The cooperative action of electromagnetic field confinement, interfacial charge transfer, and biomolecular reorganization underlies the highly sensitive detection of CA125 achieved with this SERS platform. This mechanistic understanding not only explains the spectral features observed in our measurements but also highlights the potential of plasmonic semiconductor–metal nanohybrids for probing structural and conformational details of clinically relevant protein biomarkers.

Figure 8 presents the linear fit of the SERS intensity of the 829 cm^–1^ band as a function of CA125 antigen concentration, demonstrating very good linearity. Analysis of the SERS intensity behavior gave a limit of detection of 14 U/mL, which is useful considering that 35 U/mL is the cutoff concentration used in ovarian cancer diagnosis.^6^

**Figure 8:**
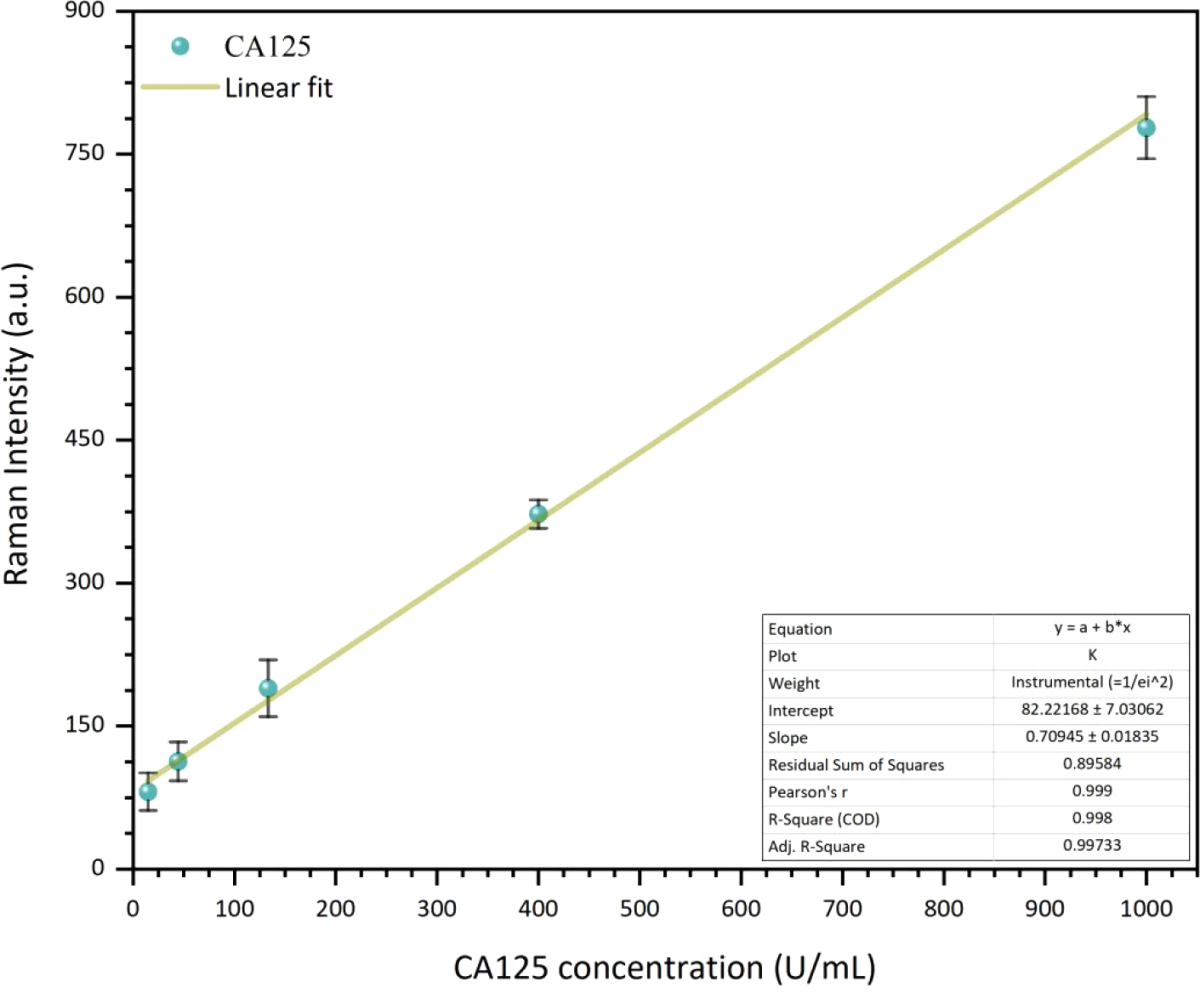
Linear fit of the SERS intensity of the 829 cm^–1^ vibrational mode versus the CA125 concentration.

To validate the performance of the SERS platform, we compared its results with those obtained by a conventional ELISA assay under identical experimental conditions. Figure 9a shows the absorbance behavior versus the CA125 concentration obtained by ELISA. Figure 9b illustrates the Raman intensity behavior of the 829 cm^–1^ vibrational mode as a function of the CA125 content as determined by SERS. A similar behavior is observed for the range from 10 to 1000 U/mL, indicating that the bioconjugation for CA125 detection has been satisfactorily developed.

**Figure 9:**
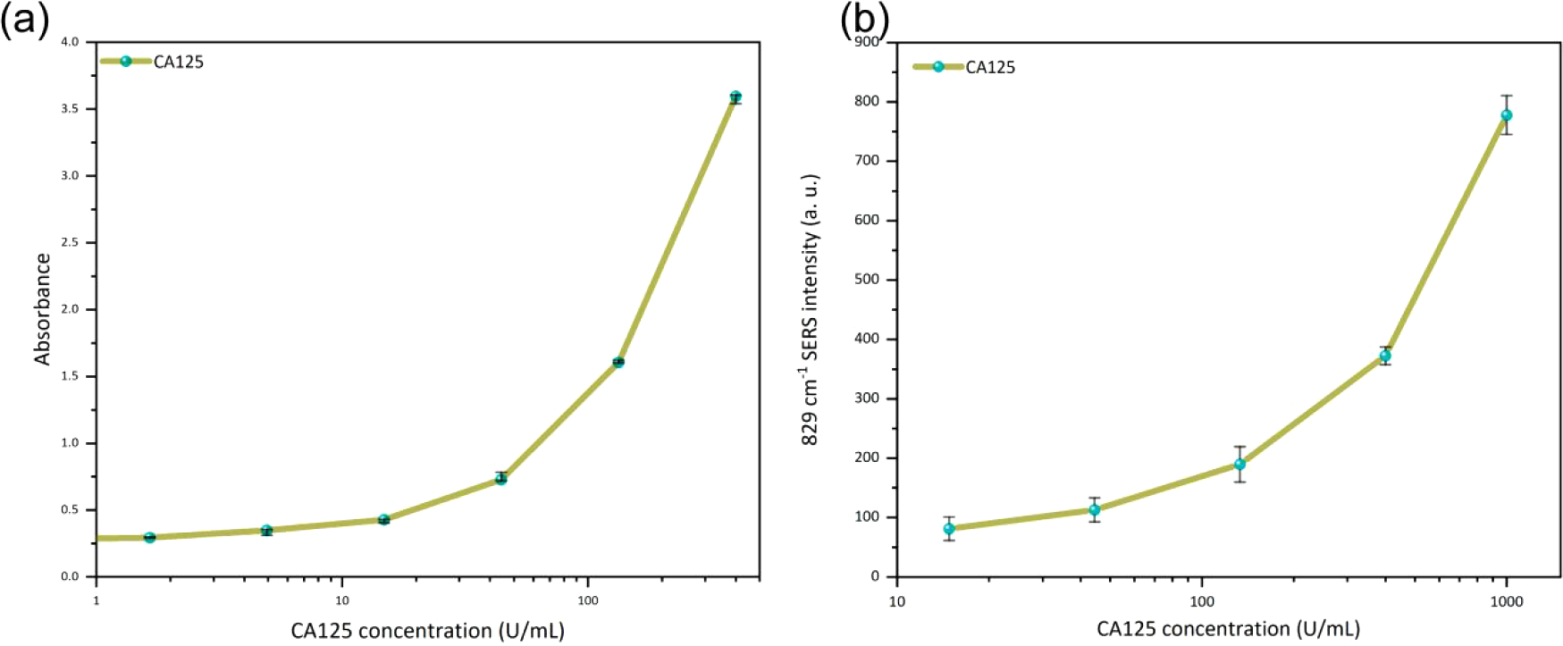
Behavior of absorbance on the ELISA study (a) and 829 cm^–1^ SERS intensity (b) versus CA125 concentration.

In summary, the results strongly suggest that the Raman signal is predominantly influenced by the antibody–antigen interaction, providing spectroscopic information that reflects key molecular features of the complex and allowing the identification of characteristic vibrational signatures associated with tyrosine and proline residues. The three-dimensional Ag/ZnO nanorod architecture markedly enhances plasmonic coupling and detection sensitivity, enabling performance relevant to clinical diagnostic requirements. These findings, including molecularly informative spectral features, improved biomolecule–surface interactions, and compatibility with standard immunoassays, highlight the potential of this approach as a promising alternative to previously reported CA125 detection strategies. In addition, by eliminating the need for external Raman labels and minimizing functionalization steps, the assay simplifies the workflow, reduces cost and variability, and provides features that are highly desirable for future translation into clinical diagnostics and point-of-care testing.

## Conclusion

The performance of a SERS substrate based on ZnO nanorods decorated with silver nanoparticles was evaluated. The substrate was optimized for the silver photoreduction time, functionalization, and surface activation with MPA and EDC/NHS. The vibrational modes generated by anchoring the CA125 antibodies on the optimized substrate were studied by SERS measurements. Finally, when the CA125 antigen was bound using a washing protocol similar to the ELISA approach, a SERS signal at 829 cm^–1^ (mainly associated with tyrosine and proline) was identified, which correlated with the concentration of CA125 and allowed the construction of a calibration curve similar to that of the ELISA. Additional studies are still required to fully understand why the signals originating from these amino acids dominate over others; nevertheless, this work demonstrates a novel label-free SERS assay for CA125 detection. Moreover, the tyrosine vibrational mode has also been observed in label-free Raman measurements of blood serum samples from patients diagnosed with ovarian cancer using other techniques,^81–83^ which suggests that the SERS substrate developed in this work could be highly valuable for obtaining molecular information on biomarkers in clinical samples. Building on this proof-of-concept, future work will aim to evaluate the platform’s performance in more complex and clinically relevant biological matrices, such as serum or plasma. This next stage will be essential to validate the practical applicability of the developed substrate in real diagnostic environments.

## Supplementary Material



## Data Availability

All data supporting the findings of this study are included in this article.

## Notes

Information on SEM images about vertical linearity of ZnO NRs and AgNPs photoreduction; SERS performance evaluation with R6G and Ab CA125 (PDF)
