# Supplementary material for: SERS-Based Immunoassay on Ag/ZnO Nanorod Substrates for Detection of CA125 Antigen
Source: ACS Meas Sci Au. 2025 Dec 2;6(1):46–58. doi: 10.1021/acsmeasuresciau.5c00108 (PMC12921610; doi:10.1021/acsmeasuresciau.5c00108)
Supplement: Supplementary file 1 [file tg5c00108_si_001.pdf]

Supplementary Information

SERS-based immunoassay on Ag/ZnO nanorods substrate for detection of CA125 antigen

Luis Zamora-Peredo[1]*, María Guadalupe Soriano-Rosales[1], Adriana Baez-Rodríguez[1], Julián Hernández Torres[1], Leandro García-González[1], Marcos Luna Cervantes[1], Enrique Juárez-Aguilar[2]

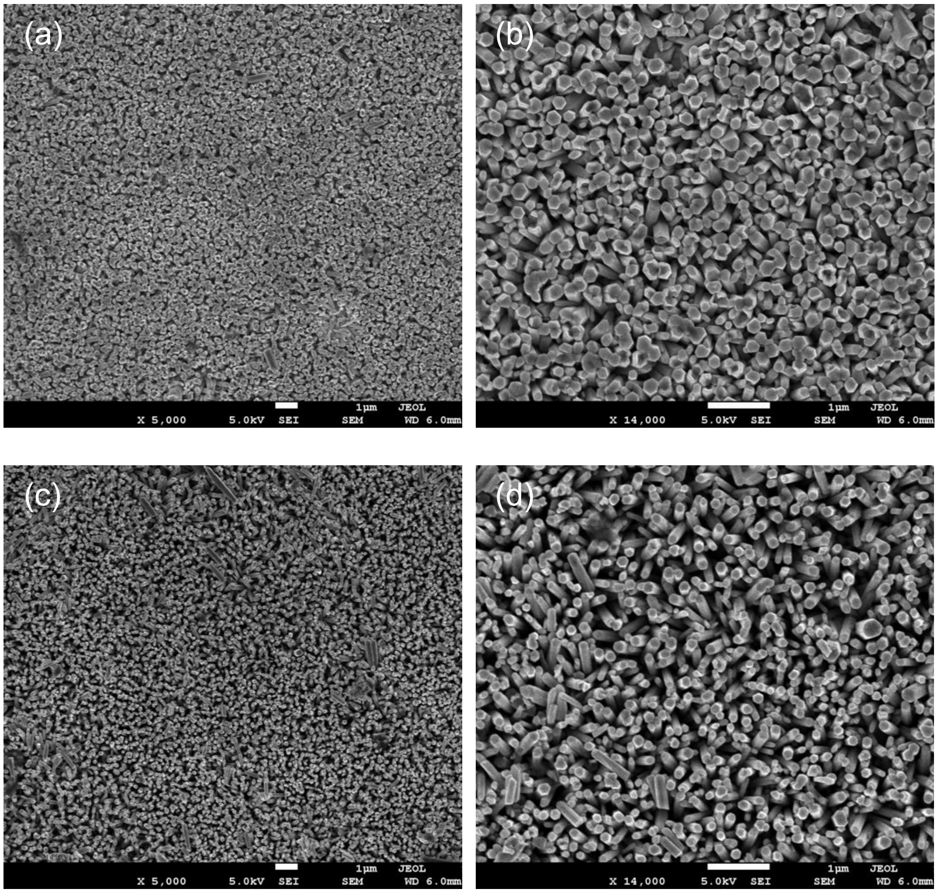

Figure S1. SEM images of ZnO NRs with good (a, b) and bad (c, d) vertical linearity.

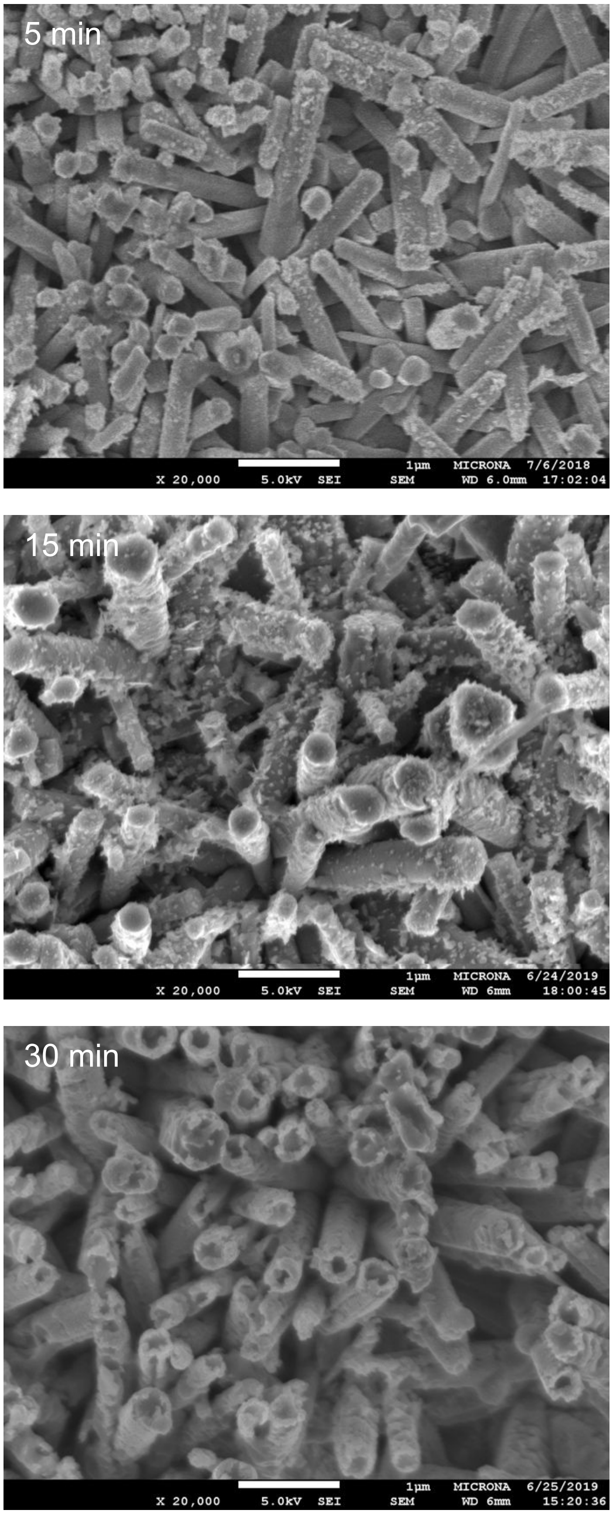

Figure S2. SEM images of ZnO NRs after 5, 15, and 10 min of AgNPs photoreduction

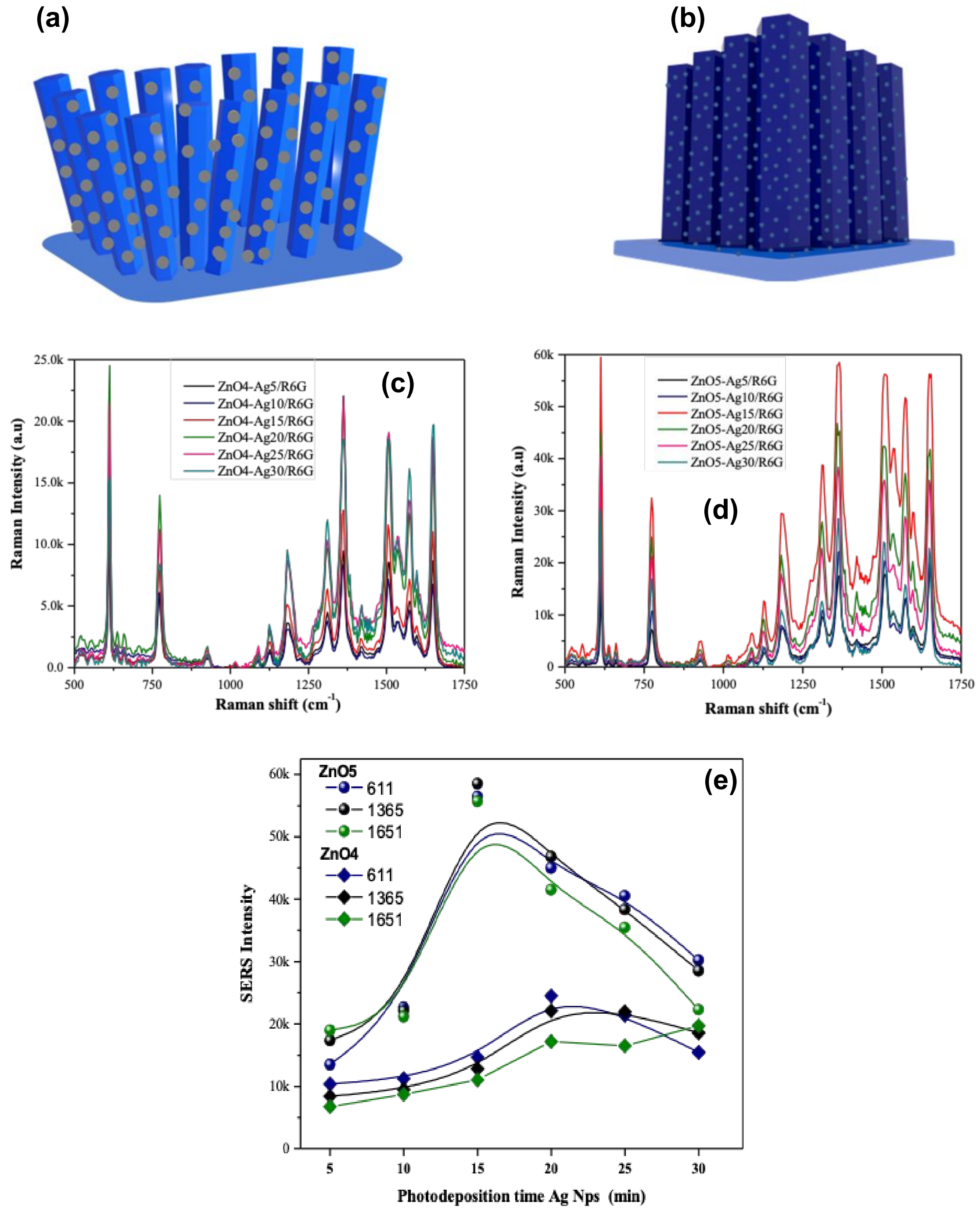

Figure S3. Schematic of ZnO NRs with “bad” (a) and “good” (b) vertical linearity and its respective Raman evaluation with $1x10^{-3}$ M R6G after AgNPs photoreduction during 5-30 min range (c, d) and behavior of SERS intensity of vibration modes versus photoreduction time.

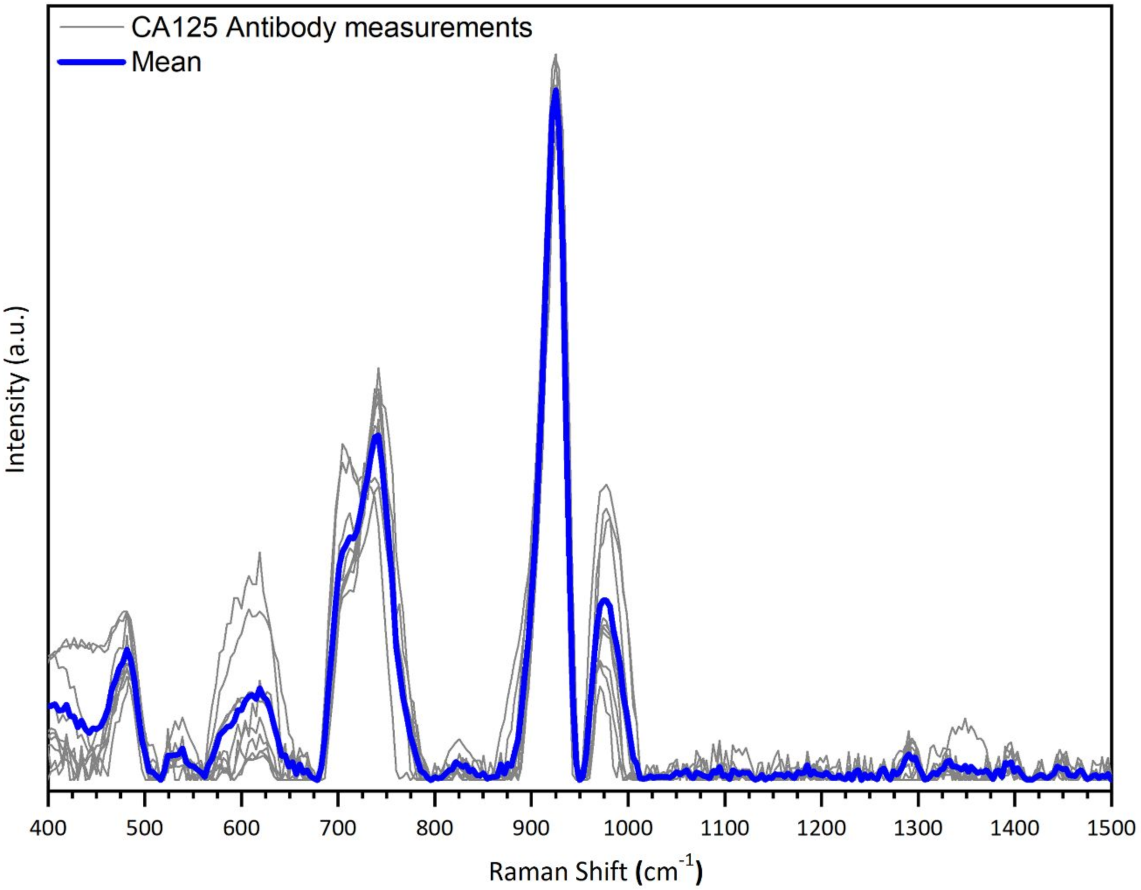

Figure S4. SERS spectra obtained from ten independent measurements of the CA125 antibody at a concentration of 0.01 mg/mL on the functionalized substrate. The gray lines represent individual spectra, while the blue line corresponds to the mean spectrum, confirming the homogeneity of the functionalized surface.
